# Increasing Insulin Pump Use in Youth with Type 1 Diabetes While Addressing Disparities

**DOI:** 10.1097/pq9.0000000000000946

**Published:** 2026-10-28

**Authors:** Kimberly K. Vidmar, Rachel J. Fenske, Whitney Beaton, M. Tracy Bekx, Elizabeth A. Mann

**Affiliations:** From the * Division of Pediatric Endocrinology and Diabetes, Department of Pediatrics, University of Wisconsin School of Medicine and Public Health, Madison, Wis.; † Department of Clinical Nutrition, UW Health, Madison, Wis.; ‡ Division of Pediatric Endocrinology and Diabetes, Department of Pediatrics, UW Health Kids, Madison, Wis.

## Abstract

**Introduction:**

Automated insulin delivery is recommended for youth with type 1 diabetes (T1D). We aimed to increase the percentage of youth with T1D (diagnosed > 1 y) in 1 pediatric health system actively using an insulin pump from 56% to 65% within 18 months while reducing disparities based on race/ethnicity and insurance type.

**Methods:**

Following the Model for Improvement, local stakeholders organized this quality improvement project, identified a theory of change, and tested change ideas using the plan-do-study-act framework. We evaluated the primary outcome with a statistical process control chart, secondary outcomes with run charts, and other measures with descriptive statistics.

**Results:**

Between February 2024 and August 2025, insulin pump use increased from 56% to 65%, but disparities by race/ethnicity and insurance type persisted. Time from insulin pump education to initiation decreased by 19.8 days for patients who attended a group technology class. Non-face-to-face encounter volume decreased from 938 to 862 encounters per month, and diabetes-associated ketoacidosis events remained stable at 2% per month.

**Conclusions:**

This initiative increased insulin pump use among both reference and minoritized groups of youth with T1D at a single institution and improved the efficiency of the insulin pump initiation process without a measurable increase in workload or diabetes-associated ketoacidosis events. Although increased pump adoption among both groups is encouraging, continued advocacy at the individual and policy levels is needed to achieve optimal pump use and reduce disparities.

## INTRODUCTION

Automated insulin delivery (AID) systems are recommended for youth with type 1 diabetes (T1D) and improve hemoglobin A1c (HbA1c), time in range, and quality of life while reducing diabetes complications.^1–10^ An AID system integrates a continuous glucose monitor (CGM), insulin pump, and control algorithm to calculate and adjust insulin delivery in real time to maintain glucose levels within a target range. Despite American Diabetes Association *Standards of Care in Diabetes* recommendations that AID is the preferred insulin delivery method for T1D, adoption remains suboptimal.^10,11^ Quality improvement and the learning health system framework could narrow the gap between evidence-based recommendations and real-world practice.^11^

Disparities in AID system use based on race and ethnicity and health insurance type are well described nationally, with higher usage in non-Hispanic White individuals as well as those with private health insurance.^5,8,12–15^ Barriers to AID use include financial burden, insurance coverage, physical interference and discomfort, concerns about therapeutic effectiveness and safety, language and health literacy, variable prescribing practices, and provider bias.^15,16^ As of February 2024, only 56% of patients seen at UW Health Kids were using an insulin pump, with lower uptake among those with public health insurance (23% lower than privately insured) and those who identify as Hispanic or non-Hispanic Black (29% lower than the non-Hispanic white population).

Other institutions have used quality improvement initiatives to increase pediatric insulin pump use and improve the pump start process, but few have focused on disparities.^17–19^ A recent study by Vakharia et al showed increased insulin pump use among youth younger than 18 years of age with recently diagnosed T1D (<1 y) receiving care at a freestanding, tertiary pediatric hospital, without an increase in diabetes-associated ketoacidosis (DKA) events, but it did not specifically address disparities.^19^ Our study addresses known disparities by testing locally tailored strategies to reduce gaps in diabetes technology use.

Our global aim was to improve glycemic outcomes and quality of life among pediatric patients with T1D. Our specific aim was to increase the percentage of monthly visits to our diabetes clinic in which individuals with T1D (diagnosed > 1 y) are actively using an insulin pump from 56% to 65% between February 2024 and August 2025 while reducing disparities in use by race/ethnicity and insurance type. Because all currently available US Food & Drug Administration–approved pediatric insulin pumps can function as AID systems and all pumps in our practice are used as AID systems, we use “insulin pump” as a proxy for AID throughout this article.

## METHODS

### Context

UW Health Kids is a tertiary pediatric health system within UW Hospital and Clinics in Madison, Wis. UW Health Kids Pediatric Diabetes Clinics provide care for approximately 900 youth ages 0–24 years with T1D annually across three clinic sites. Approximately 80% reside in Wisconsin, 20% in Illinois. Approximately 15% self-identify as a race other than non-Hispanic white. Roughly 30% are enrolled in Medicaid (~20% in Wisconsin Medicaid, and ~10% in Illinois Medicaid). Before this project, the local process for starting an insulin pump varied. After discussion with a provider, most youth met separately with a certified diabetes care and education specialist (CDCES) in person to receive initial education, participate in shared-decision making about insulin pump selection, and initiate an insulin pump ordering. Occasionally, nurse practitioners provided this training during regular diabetes follow-up visits or during separate education visits. Limitations included part-time CDCES staffing (0.6 full-time equivalent), communication challenges (eg, limited clinical context from the referring provider), unclear expectations among stakeholders, and variable training and follow-up.

### Interventions

Since 2022, UW Health Kids Pediatric Diabetes has participated in the T1D Exchange Quality Improvement Collaborative (T1DX-QI). This learning health system brings together pediatric and adult diabetes centers across the United States to collaborate and share experiences to improve care and outcomes for people living with diabetes. In February 2024, our existing T1DX-QI workgroup formed a stakeholder team of two pediatric endocrinology faculty members, one pediatric endocrinology fellow, one registered dietitian, and one clinical nurse specialist. The CDCES and ambulatory diabetes nurses served as consultants throughout the project, providing front-line knowledge and experience to the project team and serving as early adopters during intervention testing. Applying the Institute for Healthcare Improvement’s Model for Improvement, the workgroup met regularly to identify goals, identify key drivers (Fig. 1), develop change ideas, and test interventions using the plan-do-study-act (PDSA) framework. We conducted the following PDSA ramps:

**Fig. 1.**
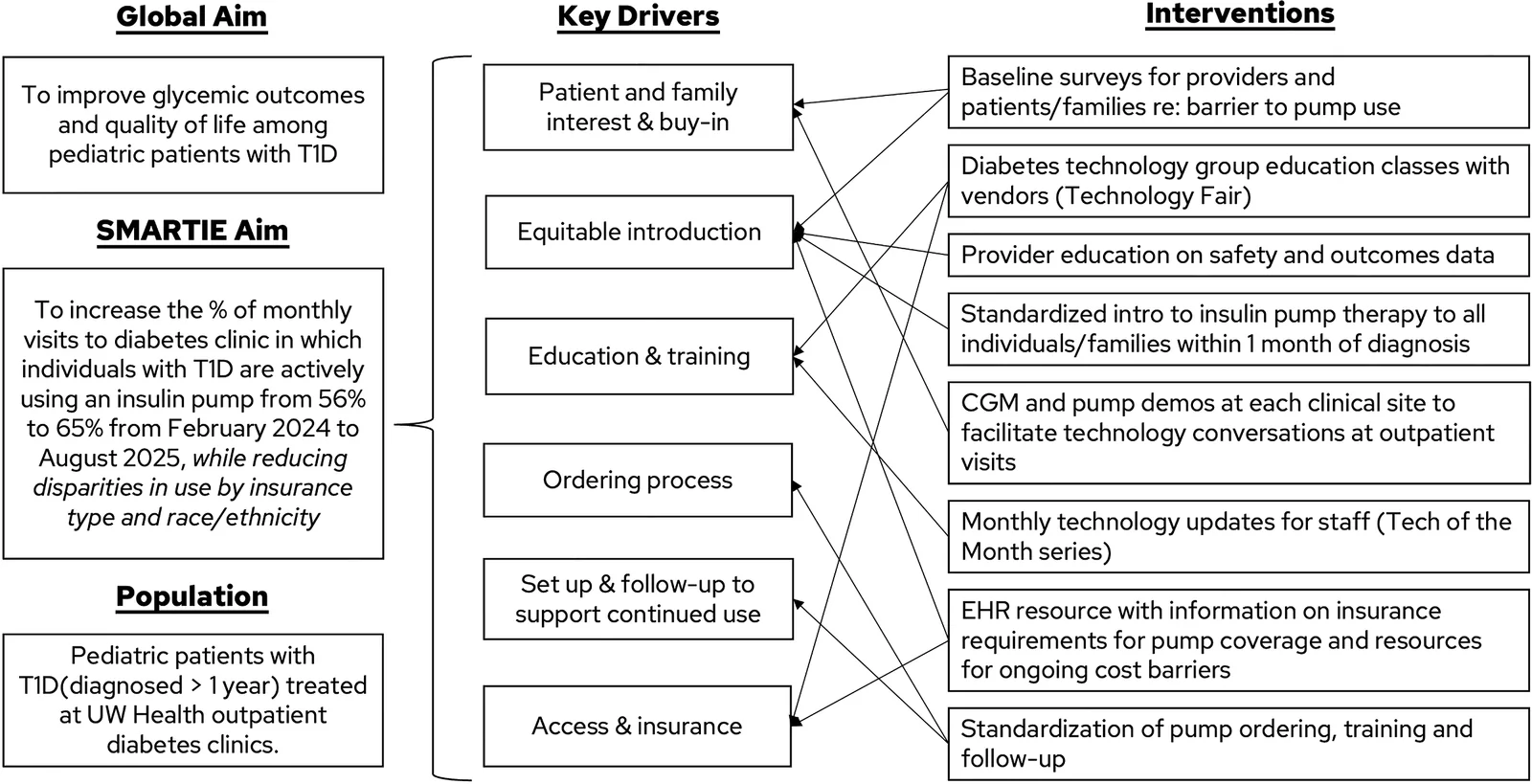
Key driver diagram.

1. **Baseline surveys:** In March 2024, we surveyed patients, caregivers, and providers about perceived barriers to pump use (see Appendices A and B, **Supplemental Difigtal Content 1,** https://links.lww.com/PQ9/A841.
  We invited patients and caregivers of patients not using pumps who attended in-person clinic from March–May 2024 to complete a paper survey. All ambulatory diabetes staff and providers received a Qualtrics (Provo, Utah) survey that also assessed prescribing practices. Patients and caregivers identified wearability as the predominant barrier, whereas providers emphasized wearability, insurance coverage, and trust in technology. Providers also reported variable prescribing practices influenced by HbA1c, time since diagnosis, and insurance. Findings raised awareness of bias and informed key drivers and PDSA cycles.
2. **Technology fair:** Beginning in May 2024, we developed and iterated a recurring, in-person, group program to support children with T1D and their caregivers in learning about insulin pump therapy. Members of the diabetes care team referred patients regardless of time since diagnosis or current diabetes management modality. We shared information about the course through discussion and provided a flyer. Families were initially scheduled by completing an electronic survey, and we later integrated scheduling into the electronic health record (EHR; Epic Systems, Verona, Wis.). Families received a folder with reference materials, including a note-taking sheet for class entry. A registered nurse and a CDCES led the first 60 minutes of structured education using a PowerPoint presentation including insulin pump fundamentals, technology troubleshooting, safety considerations, available pumps, and an overview of the insulin pump initiation process at UW Health Kids. During the second 60 minutes, representatives from each insulin pump company with an FDA-approved pediatric pump (Insulet Corporation [Omnipod 5; Acton, Mass.], Tandem Diabetes Care [t:slim X2 and Mobi System; San Diego, Calif.], Beta Bionics [iLet Insulin Pump; Irvine, Calif.], Medtronic Corporation [MiniMed 670G and MiniMed 780G; Minneapolis, Minn.]) were invited to provide device demonstrations and answer product-specific questions. Sessions averaged 8–10 patients and increased from monthly to 2–3 times per month due to demand. After observing variable expectations and communication styles among families during the pump start process, we modified the patient education materials to include a general overview and timeline. We scheduled a follow-up phone call with our CDCES for each patient in attendance to allow time for patient-specific questions, identify knowledge gaps, and begin the pump ordering process, if desired.
3. **Provider education to address perceived barriers**: In August 2024, we held a one-time, 60-minute virtual provider education session to address implicit bias and variable prescribing practices identified in baseline surveys. All pediatric diabetes providers attended, including 8 pediatric endocrinology faculty, 3 pediatric endocrinology fellows, and 2 pediatric diabetes nurse practitioners. QI workgroup members led the session, which reviewed local pump use and disparities, evidence on benefits and safety, and persistent technology inequities, then discussed prescribing inconsistencies, potential selection bias, and barriers to pump use.
4. **Standardized introduction at diagnosis:** We created patient education materials introducing insulin pump basics and disseminated these to all patients and caregivers at the time of T1D diagnosis starting in October 2024.
5. **Pump demos:** In October 2024, our team provided each UW Health Kids Peds Diabetes clinic with demos of all available FDA-approved insulin pump systems and CGMs to facilitate technology discussions with patients in real time. We educated providers and nursing staff about their availability and encouraged use through frequent emails, reminders during team meetings, and clinic signage.
6. **Tech of the Month series:** In January 2025, we began this educational initiative designed to enhance staff and clinician proficiency with CGM and insulin pump systems. Each month highlights a specific device and includes two 1-hour live training sessions facilitated by local industry representatives and clinical trainers to maximize staff access. To reinforce content or share information with those who could not attend, the clinical nurse specialist disseminated materials throughout the month, including a monthly summary email. These resources highlighted knowledge on ordering, technical specifications, key clinical features, and data sharing.
7. **Financial resources:** We created a compilation of financial resources for patients and families exploring insulin pumps in June 2025 and shared it with the entire clinical team, including providers. We developed this quick EHR resource, including links to financial resources by insurance type, to address providers’ concerns and reduce cost barriers for families.
8. **Standardizing pump orders and training**: We created automated text tools within the EHR outlining insulin pump ordering, pump initiation, and follow-up to streamline internal communication and delegate tasks.

### Measures and Data Collection

The primary outcome measure was the percentage of pediatric diabetes clinic visits in which the individual with T1D (diagnosed > 1 y) is actively using an insulin pump. Our team selected this population because these data are reported monthly via secure data transfer to a cloud-based server associated with the T1DX-QI Coordinating Center and shared back with our center via a secure online portal.^20^ We analyzed the outcome measure using statistical process control via a P-chart to identify trends and changes over time. The same online portal stratified the outcome measure by self-reported race and ethnicity and health insurance type. We compared the percentage of visits with active insulin pump use between the reference and minoritized groups using run charts.

Process measures included the annual number of insulin pump starts, Technology Fair attendance per session, the time from insulin pump education to pump start, and the time from insulin pump order to insulin pump start. We collected these metrics via manual EHR review and reported them using descriptive statistics.

For balancing measures, we sampled the monthly telephone call volume and patient messages via the EHR (eg, MyChart messages in Epic) and reported them collectively as non-face-to-face encounters. We also tracked the monthly percentage of patients with T1D who were admitted with DKA using the T1DX-QI portal.

### Ethical Considerations

This work represents a quality improvement project and does not constitute research as defined under 45 CFR 46.102(d); therefore, it did not require formal institutional review board review under federal regulations.

## RESULTS

Between February 2024 and August 2025, the percentage of patients with T1D (diagnosed >1 y) actively on an insulin pump increased from 56% to 65% (Fig. 2), meeting our initial aim. Starting in October 2024, 8 consecutive points fell above the mean centerline, suggesting special cause variation and shifting the mean centerline to 65%. In the 6 months following our active study period, we observed no special cause variation in either direction.

**Fig. 2.**
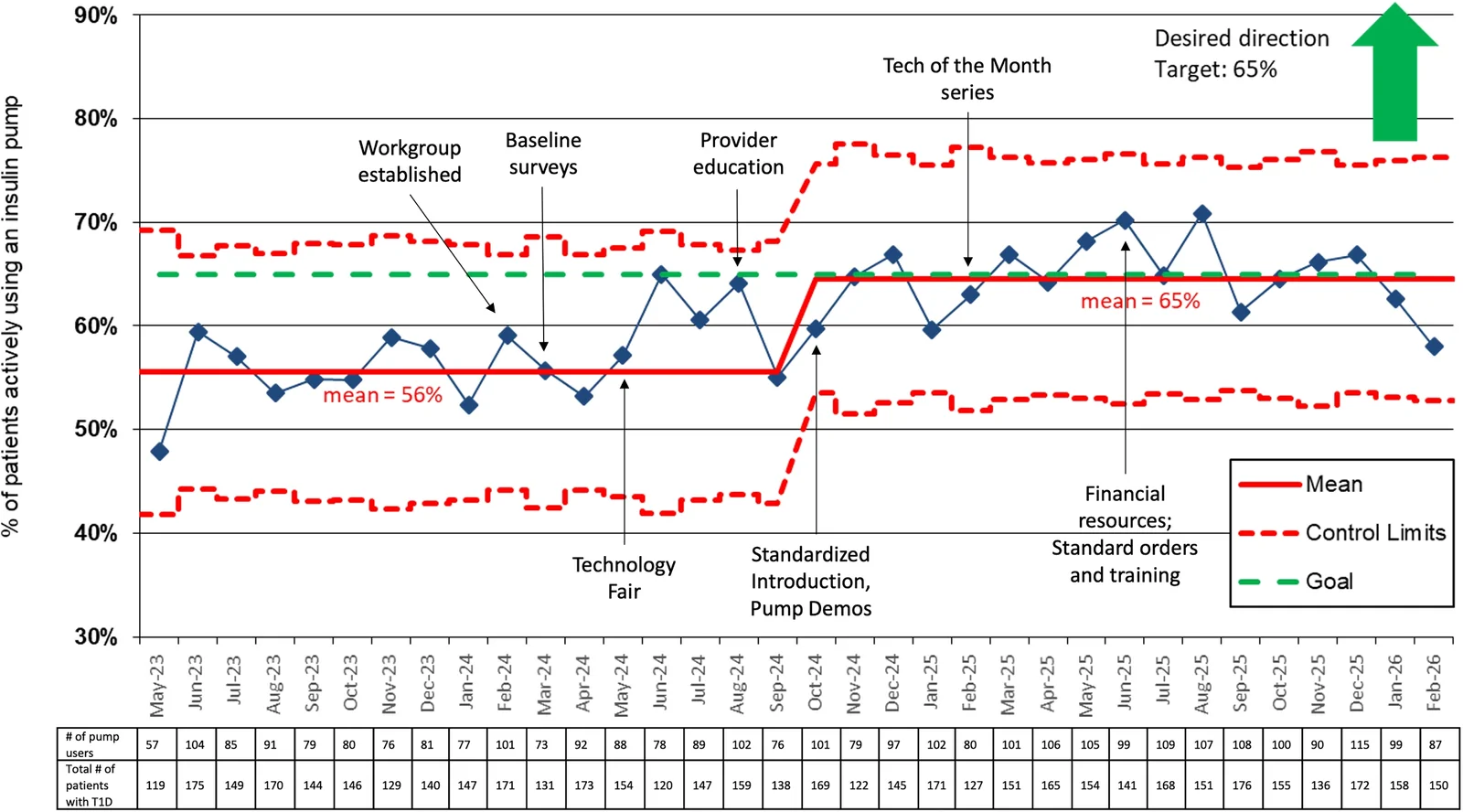
Percentage of monthly visits during which an individual with T1D (diagnosed >1 y) is actively using an insulin pump.

The disparity in insulin pump use between non-Hispanic white and Hispanic and non-Hispanic Black youth varied throughout the study period, ranging from approximately 14%–64% (Fig. 3A). Although it occasionally fell below the median of 44%, evidence of sustained special cause improvement remains insufficient. Similarly, the disparity in insulin pump use between privately insured and Medicaid-insured youth persisted, with monthly differences ranging from approximately 14%–52%. Starting in October 2024, privately insured patients showed eight consecutive points above the median centerline, indicating special cause variation and prompting a shift in the median centerline from 70% to 75%. Similarly, beginning in February 2025, patients with Medicaid showed eight consecutive points above the median centerline, indicating special cause variation and prompting a shift in the median centerline from 38% to 45% (Fig. 3B). Overall, the disparity narrowed modestly by 2%, but a gap in insulin pump use persisted.

**Fig. 3.**
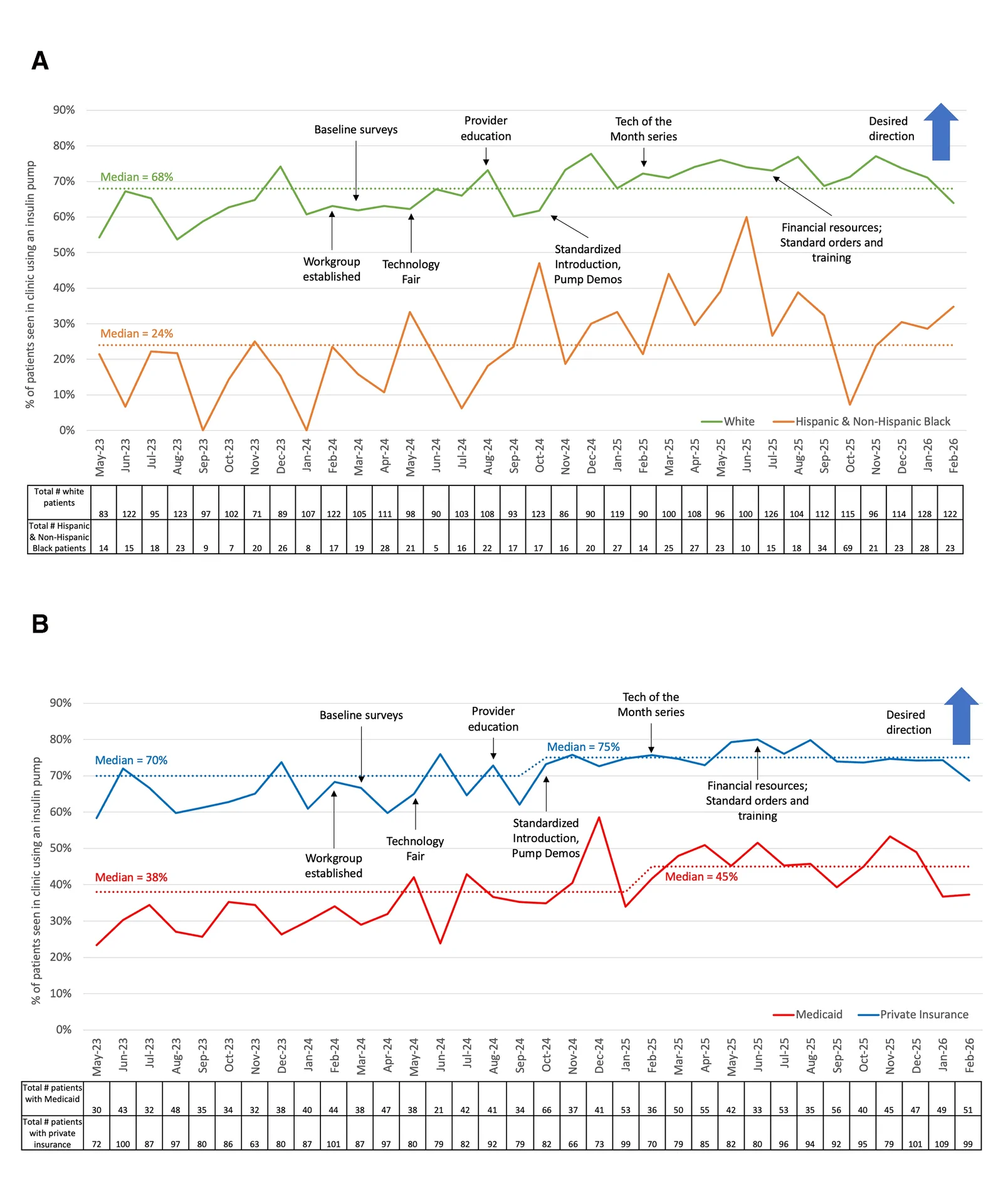
A, Run chart demonstrating the percentage of individuals with T1D (diagnosed >1 y) actively using an insulin pump who self-identified as White (green) and Hispanic and/or non-Hispanic Black (orange). B, Run chart demonstrating the percentage of individuals with T1D (diagnosed >1 y) using an insulin pump with private insurance (blue) and Medicaid (red).

### Process Measures

Annual pump starts increased after we initiated our quality improvement efforts. There were 87 pump starts in 2023, 122 in 2024 and 123 in 2025. Most patients who attended a Technology Fair subsequently ordered an insulin pump (57/70, 81%) and started insulin pump therapy (51/70, 73%). The time elapsed from insulin pump education to insulin pump start (lead time) was 19.8 days shorter for patients who attended a Technology Fair than for those who attended a one-on-one technology visit. Finally, time from pump order to pump start was shorter for devices available as a pharmacy benefit (ie, Omnipod 5) than for those only available through a durable medical equipment (DME) third party or the pump company itself (Fig. 4).

**Fig. 4.**
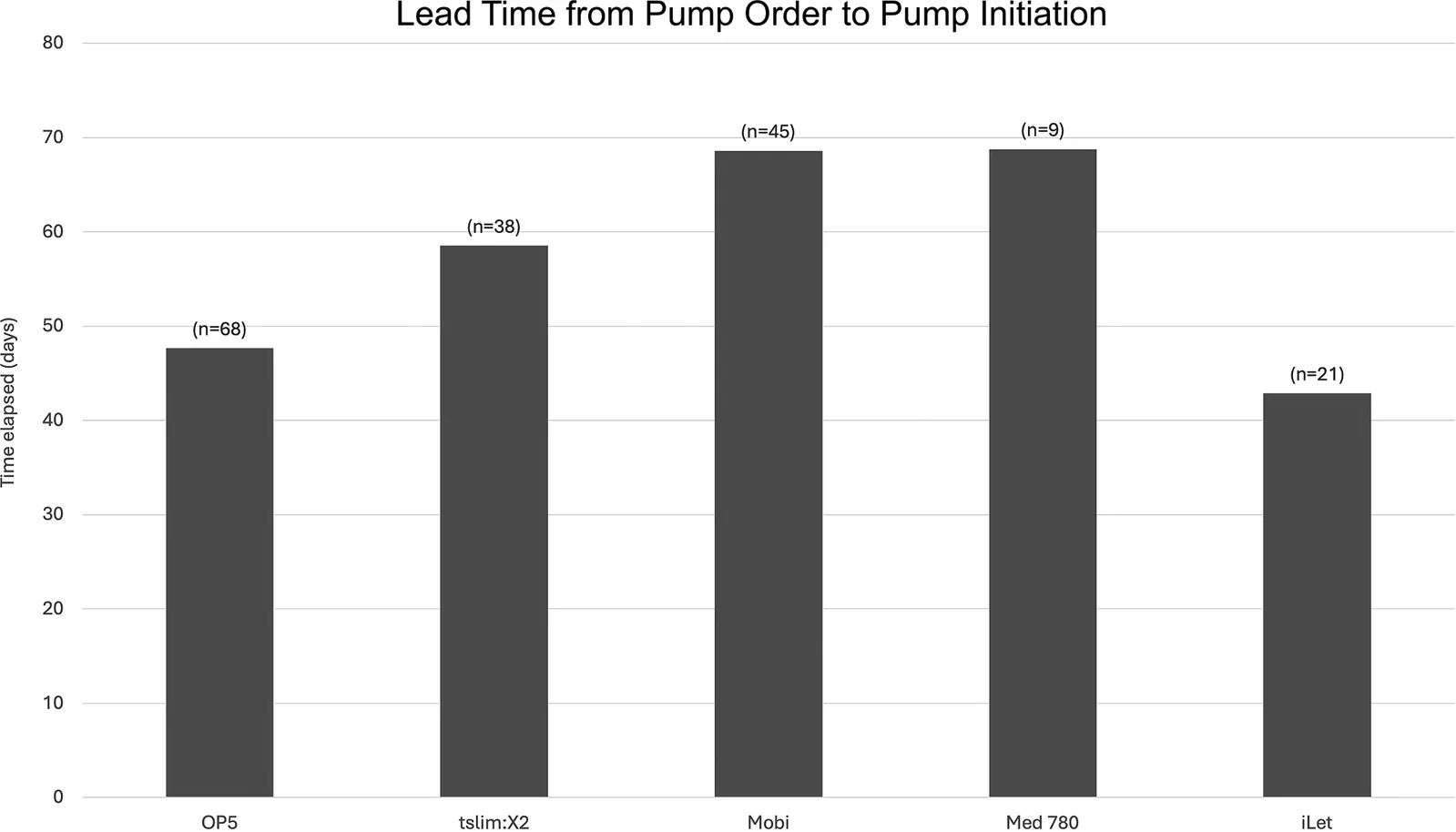
Lead time from pump order placement to pump initiation from January 2024 through April 2025 stratified by pump brand. iLet, Beta Bionics iLet; OP5, Omnipod 5; Mobi, Tandem Mobi; Med 780, Medtronic MiniMed 780G; tslim:X2, Tandem t:slim X2.

### Balancing Measures

The average number of non-face-to-face encounters (ie, MyChart messages and telephone calls) was 938 per month before implementation of the Technology Fair and 862 per month in the 12 months after implementation. Average monthly DKA rates remained stable at 2% throughout the project.

## DISCUSSION

This initiative increased insulin pump use among pediatric patients with T1D at a single institution without increasing unscheduled clinic contact or DKA events while shortening time from pump education to initiation. Forming a multidisciplinary workgroup and systematically identifying barriers to pump use through baseline surveys raised awareness of suboptimal pump use rates and existing disparities, likely contributing to improvement in pump use rates independent of subsequent interventions. Expanding access to diabetes technology education for patients and their families through the Technology Fair was integral to improving access to insulin pump therapy, given our center’s limited CDCES resources. Offering group pump education through the Technology Fair also decreased time from education to pump ordering and likely contributed to an increase in annual pump starts.

This work highlights several practical interventions that other centers with similarly limited diabetes education resources can readily adapt and implement. In particular, the successful implementation of the Technology Fair underscores the value of partnering with industry representatives to enhance and streamline insulin pump education, increase patient exposure to available technologies, and reduce the educational burden on clinical teams.

Given known local and national disparities in diabetes outcomes and technology utilization by race/ethnicity and insurance type, the T1DX-QI Equity Framework guided our efforts to close this gap.^21^ Despite multiple interventions targeting disparities in pump use (expanded technology education, addressing provider bias, standardized introduction, financial resources), insulin pump use remained lower among non-Hispanic Black or Hispanic patients and those with public insurance. It is unclear whether these racial disparities would have persisted after controlling for insurance, given the limitations of our data collection and our small sample size, although prior studies show that they remain even after adjustment for insurance and socioeconomic factors.^15^ Although our interventions addressed access to and availability of education through the Technology Fair, this did not eliminate barriers related to health literacy, language, transportation, or time away from work.

Additionally, although the financial resources were easy to disseminate and implement, they did not address the systemic inequities faced by patients with public insurance. Publicly insured patients from Wisconsin face unique and significant barriers to accessing diabetes technology. First, Wisconsin Medicaid does not cover CGMs as a pharmacy benefit and requires a DME vendor, whereas almost all private insurance policies cover CGMs as a pharmacy benefit. This increases the burden on patients and families with Wisconsin Medicaid to coordinate prescriptions, insurance information, shipping address, and refills over the phone. Second, Wisconsin Medicaid excludes coverage for insulin pumps available only through a pharmacy benefit (eg, Insulet’s Omnipod 5). This eliminates choice based on form factor, as the Omnipod 5 is the only tubeless insulin pump available in the United States. The insulin pump ordering channel creates disparities in use and in time to access pump therapy. For patients with private insurance who chose an insulin pump available through DME (eg, Tandem’s t:slim X2 and Mobi or Medtronic’s MiniMed 670G/780G), the time from insulin pump order to start was longer for nonpharmacy-benefit pumps. These delays affect the patient experience and can create confusion and distrust in the system. State-level advocacy will remain vital to reducing disparities in diabetes technology use and providing equitable access to standard-of-care insulin pump therapy for all patients.

To inform the application of lessons learned and the dissemination of this work to other centers, we highlight key limitations. The primary outcome measure included only patients diagnosed with T1D for >1 year, as the T1DX-QI portal reported data this way at the time. Patients with new-onset T1D are an especially important population to target for early pump initiation, and our data may underestimate the potential impact of standardized pump introduction; this exclusion limits our study’s generalizability. Additionally, the outcome measure included only patients attending in-person clinic visits, underrepresenting those with fewer or no visits and potentially introducing measurement bias. Population-level insulin pump use is therefore an important measure to track alongside the more dynamic measures. Given the real-world nature of this work, another limitation is the representation of T1D patients from minoritized populations. We attempted to address the small sample size in each racial and ethnic group by combining groups to compare insulin pump use with the non-Hispanic white group. Larger, multicenter quality improvement work through the T1DX-QI is needed to explore how targeted interventions affect disparities in use. This project did not measure pump discontinuation rates beyond overall use rates nor did it explore reasons patients may stop using an insulin pump. Ongoing work includes developing a patient and family education curriculum on pump optimization to empower users to use the technology efficiently, avoid complications, and prevent interruptions or discontinuation.

Although it is encouraging that insulin pump uptake increased among both reference and minoritized groups of youth with T1D, further work is needed to achieve optimal insulin pump use rates and ultimately reduce disparities. We recognize that multilevel barriers drive disparities in diabetes technology use and will require multilevel solutions that extend beyond the scope of this single-institution quality improvement initiative. Achieving equitable access will require sustained advocacy at both the individual and policy levels. Reducing administrative and insurance-related barriers, particularly those tied to DME procurement pathways, represents an important opportunity to improve access to insulin pump therapy and advance health equity for people with T1D.

## ACKNOWLEDGMENTS

The authors thank Tricia Griffin, Juliana Price, MSN, and the rest of the UW Health Kids Pediatric Diabetes Team for their ongoing support, collaboration and dedication to this quality improvement work and our patients. The authors thank Jack Stevens, PhD, for his mentorship, thoughtful review of the manuscript, and valuable feedback throughout the development of this work.

## Supplementary Material
