# Supplementary material for: Increasing Insulin Pump Use in Youth with Type 1 Diabetes While Addressing Disparities
Source: Pediatr Qual Saf. 2026 Oct 28;11(6):e946. doi: 10.1097/pq9.0000000000000946 (PMC13716418; doi:10.1097/pq9.0000000000000946)
Supplement: Supplementary file 1 [file pqs-11-e946-s001.pdf]

Appendix A – Patient Survey

Barriers to Insulin Pump Use

The UW Heath Kids Diabetes Team needs your input! We want to learn more about why you or your child may NOT be currently using an insulin pump. The survey will not ask your name, so it is anonymous. Please complete the survey below OR scan the QR code with your smart device to complete. . If you would like to learn more about insulin pumps or have questions about this survey, please ask your diabetes team today.

Please check all the reasons you (or your child) do not use an insulin pump (you may select more than one):

- ☐ Did not know this was an option
- ☐ Nervous to rely on technology
- ☐ Do not know how to use it
- ☐ Insurance coverage - If you checked this box, what insurance do you have? ____________________
- ☐ Too expensive
- ☐ Do not like having another device on body
- ☐ Too many alarms
- ☐ It is uncomfortable or painful
- ☐ Takes too much time to use (refilling pump, change pump site)
- ☐ Do not want another device because people will notice and ask questions
- ☐ Time needed to complete pump training and set up
- ☐ Interferes with sleep
- ☐ Other reason(s): __________________________

  If you want to share anything else with us about getting an insulin pump, please share it here:

Appendix B – Provider Survey

Our T1DX QI team is working on a project to improve access to insulin pumps and automated insulin delivery systems for our patients. We are collecting input from our whole team and patients to understand where the opportunities and challenges may exist. Your feedback and time are greatly appreciated. This survey should take no more than 5 minutes of your time.

1. What is your role on the diabetes team?
   - ☐ Provider (MD, NP, PA)
   - ☐ RN, DCES, RDN, or MA
   - ☐ Other: ___________________

2. Is part of your role to prescribe insulin pumps to patients?
   - ☐ Yes
   - ☐ No

3. (If answered 'Yes' to Q2) What factors influence your choice to bring up insulin pump therapy?

4. (If answered 'Yes' to Q2) I talk about insulin pumps routinely at diagnosis in the hospital
   - ☐ All the time
   - ☐ Most of the time
   - ☐ Sometimes
   - ☐ Rarely
   - ☐ Never
   - ☐ Not applicable

5. (If answered 'Yes' to Q2) For patients not currently using an insulin pump, I talk about pumps at clinic visits
   - ☐ All of the time
   - ☐ Most of the time
   - ☐ Sometimes
   - ☐ Rarely
   - ☐ Never

□ Not applicable

6. (If answered ‘Yes’ to Q2) I have an ideal HbA1c that I would like my patients to achieve before initiating pump therapy

   □ Yes - please enter ideal HbA1c __________

   □ No

7. (If answered ‘Yes’ to Q2) I have an ideal amount of time that should pass since diagnosis before initiating pump therapy

   □ Yes - please enter amount of time ___________

   □ No

8. (If answered ‘Yes’ to Q2) Insurance coverage influences which patients I talk to about pump therapy

   □ Yes

   □ No

9. (If answered ‘Yes’ to Q8) Please explain how insurance coverage influences which patients you talk to about pump therapy

10. (If answered ‘Yes’ to Q2) What other factors influence when or which patients you choose to offer pump therapy? Check all the reasons that have contributed to your patients with type 1 diabetes not being on insulin pump therapy:

    □ Did not know it was an option

    □ Nervous to rely on technology

    □ Do not know how to use it

    □ Insurance coverage

    □ Too expensive

    □ Do not like having another device on body

    □ Too many alarms

    □ It is uncomfortable or painful

    □ Takes too much time to use (refilling pump, changing pump site, etc.)

    □ Do not want another device because people will notice and ask questions

- ☐ Time needed to complete pump training and set up
- ☐ Interferes with sleep
- ☐ Other ________________________

11. As the T1DX QI team moves forward with a quality improvement project to increase access to automated insulin delivery systems for patients, is there anything else you would like to share with the team?
